# Integrated analysis of metabolome and transcriptome reveals the cytoplasmic effects of CMS-D2 on pollen fertility resulting from disrupted lipid metabolism

**DOI:** 10.3389/fpls.2022.998203

**Published:** 2022-09-30

**Authors:** Xuexian Zhang, Meng Zhang, Liping Guo, Tingxiang Qi, Huini Tang, Yongqi Li, Zhidan Zuo, Kashif Shahzad, Juanjuan Feng, Rong Zang, Hailin Wang, Xiuqin Qiao, Jianyong Wu, Chaozhu Xing

**Affiliations:** State Key Laboratory of Cotton Biology, Institute of Cotton Research of Chinese Academy of Agricultural Sciences, Key Laboratory for Cotton Genetic Improvement, Ministry of Agriculture and Rural Affairs, Anyang, Henan, China

**Keywords:** CMS-D2, negative effects, pollen fertility, lipid metabolism, flavonoid biosynthesis

## Abstract

Using cytoplasmic male sterility of *Gossypium harknesii* (CMS-D2) is an economical and effective method to produce cotton hybrids. However, the detrimental cytoplasmic effects of CMS-D2 on pollen fertility and fiber yields greatly limit the further development of three-line hybrid cotton in China. In this study, an integrated non-targeted metabolomics and transcriptome analysis was performed on mature pollens of maintainer line NB, isonuclear alloplasmic near-isogenic restorer lines NH and SH under two environments. A total of 820 metabolites were obtained, of which lipids and lipid-like molecules were the most, followed by organic acids derivatives, phenylpropanoids, and polyketides. Transcriptome analysis revealed significantly more differentially expressed genes (DEGs) in SH versus NH both in Anyang and Jiujiang, and most of the DEGs were significantly upregulated. Further KEGG analysis showed that most DEGs were enriched in the biosynthesis of unsaturated fatty acids, phenylalanine metabolism, and phagosome. Based on the weighted gene co-expression network analysis, totally 74 hub genes were also identified, of which three transcription factors, i.e., *WRKY22*, *WRKY53*, and *ARF18* were significantly upregulated in SH and may play a negative regulatory role in pollen development by directly or indirectly regulating the jasmonic acid synthesis and signal transduction. Moreover, we found that the negative effects of CMS-D2 cytoplasm on pollen fertility were mainly due to disturbed lipid metabolism, especially the metabolic balance of unsaturated fatty acids, ultimately resulting in the decline of pollen fertility. Meanwhile, in the presence of CMS-D2 sterile cytoplasm, the cytoplasmic-nucleus interaction effects generated a substantial quantity of flavonoids involved in the fertility restoration process. This study preliminarily clarified some of the reasons for the negative effects of CMS-D2 cytoplasm on pollen fertility, and our results will provide an important theoretical reference for further breeding and improvement of three-line hybrid cotton in the future.

## Introduction

Cotton has obvious heterosis, hybrid cotton usually produces more yield than inbred parents (Chen et al., 2022), and at the same time is suitable to improve fiber quality and resistance to various insect pests (Wang, 2019). The key step in the utilization of cotton heterosis is the production of hybrid seeds at the most reasonable cost. The cytoplasmic male sterility (CMS) system is the supreme method to produce hybrid seeds. CMS line with fertile restorer line has long been applied in the hybridization process to develop superior hybrids (Kim and Zhang, 2018). Because of an important pollination measure in hybrid cotton, CMS systems such as *G. harknesii* (D_2–2_) CMS-D2 (Meyer, 1975) and *G. trilobum* (D_8_) CMS-D8 (Stewart, 1992) have more practical significance. In the process of hybridization, the fertility of CMS lines is possibly restored by various restorer genes, and the restorer gene *Rf1* from the nuclear genome of D2-2 can restore the fertility of CMS-D2 (Weaver and Weaver, 1977). Up to now, the CMS-D2 system has been widely used in cotton hybrid breeding in China. However, *Rf1* has not been cloned and isolated in cotton. In addition, detrimental cytoplasm effects influence hybrid cotton yield which further limits the wide use of the CMS-D2 system (Meyer, 1973; Zhu et al., 1998; Zhang et al., 2019a). Recent research progress in CMS crops suggests that sterile cytoplasm caused substantial genetic effects on progeny, CMS-based hybrid progeny generally produces more genetic effects than conventional hybrids, therefore detrimental effects play an imperative role in yield, and quality improvement. Cytoplasmic effects can influence different stages of vegetative and reproductive growth. Previous investigation in rice, sorghum, cauliflower, cotton, and other crops have shown that most CMS lines have detrimental effects on yield-related traits and that these effects vary among CMS sources (Young and Virmani, 1990; Moran and Rooney, 2003; Dey et al., 2013; Zhang et al., 2019a). In cotton, Meyer (1973) first reported that the cytoplasm of *G. harknesii* had a significant detrimental effect on cotton. After that, several analyses showed that detrimental effects mainly decrease the number of bolls and boll weight compared with upland cotton hybrids which led to a significant decrease in yield (Zhang et al., 1992; Zhu et al., 1998; Gill et al., 2009; Zhang et al., 2019a). In addition, the cytoplasmic effect of *G. harknesii* had obvious negative effects on pollen fertility, mainly decreased pollen activity and the amount of pollen. Especially under high-temperature stress, the pollen fertility of hybrid offspring is decreased, which led to a lower boll setting rate, and finally reduced yield of CMS-based F_1_ hybrids (Zhang et al., 2018; Zuo et al., 2022).

The development of mature and fertile pollen, along with fertile is imperative for successful fertilization. Therefore, pollen viability is a key factor affecting crop yield. The decline in pollen viability is frequently with the modification of metabolite composition (Paupière et al., 2017). Mature pollen comprises a large number of secondary metabolites which influence pollen viability and fertility. Such as fatty acids and their derivatives are important components for anther cuticles as well as pollen wall formation. Moreover, lipid metabolism is essential for plants, such as anther dehiscence and pollen maturation (Ishiguro et al., 2001; Xiao et al., 2014) and pollen hydration (Haslam et al., 2015; Zhan et al., 2018). Imbalance of lipid metabolism at anther development often leads to male sterility. To date, many lipid metabolic genes involved in anther development have been identified in CMS cotton (Wan et al., 2020). Previous studies with particular mutants, sterile lines, and biosynthetic inhibitors have shown that disrupting the dynamic changes of specific metabolites including jasmonic acid, proline, flavonoids, and other hormones are closely correlated with pollen fertility. The exogenous application of these respective metabolites partially complemented pollen fertility (Falasca et al., 2010; Zechmann et al., 2011; Mattioli et al., 2012). Jasmonates as one of the important fatty acid derivatives, regulate various physiological mechanisms and are required for regulating various transcriptional responses in plants (Khan et al., 2020). Furthermore, jasmonates influenced anther dehiscence, filament elongation, and pollen viability (Scott et al., 2004), Many studies study have witnessed that delayed dehiscence or non-dehiscence phenotypes have been found in mutants defective in JA biosynthetic-related genes such as fatty acid desaturation (*fad*) mutants (Mcconn and Browse, 1996), opr3 (mutation in 12-oxophytodienoic acid reductase) (Stintzi and Browse, 2000), delayed-dehiscence1 (*dde1*) and *dde2* (Sanders et al., 2000; von Malek et al., 2002), defective in anther dehiscence 1 (*dad1*) (Ishiguro et al., 2001), and allene oxide synthase mutants (Park et al., 2002). In addition, the contribution to pollen nutrition or signal transduction, metabolites can be used as protective agents against various environmental stresses. For instance, flavonoids can also act as scavengers of reactive oxygen species (ROS) (Ha et al., 1998). In addition, lipids, flavonoids, and polyamines also influenced the development of pollen walls, including different coat layers, such as the outer wall, endoplasmic, and sporopollenin proteins. In this way, metabolites play an essential role in abiotic stress resistance (Shi et al., 2015). Despite its importance, the existing knowledge about the role of secondary metabolites in pollen development is still limited in CMS cotton.

In recent years, the frequency of extremely high-temperature has increased due to abrupt climate changes. In particular, the highest temperature is close to 40°C from July to August in the cotton zone of the Yangtze River Basin. This temperature extreme is not conducive to anther powder dispersion, affects the boll setting rate, and leads to yield reduction in CMS cotton (Zhang et al., 2018). At present, the research on the cytoplasmic effect of *G. harknessii* cotton mostly focuses on the main economic traits such as yield and quality and exclude the interaction effect of cytoplasm under different environment. Interestingly, the results of our previous field trials revealed that sterile cytoplasm had significant negative effects on cotton anther development under high-temperature stress, and the pollen fertility of sterile cytoplasmic restorer lines was significantly reduced, finally resulting in a significant decrease in cotton yield (Zuo et al., 2022).

This study was designed to better understand the molecular mechanism of pollen fertility in CMS-D2 cotton. An integrated non-targeted metabolomics and transcriptome analysis was performed to compare mature pollens of two different cytoplasmic backgrounds CMS cotton lines under different environments. The primary objective was to explore the influences of *G. harknessii* cytoplasm on pollen fertility and to establish a platform for the discovery of the molecular mechanism of the cytoplasmic sterility of *G. harknessii* cotton. The results of our study offer a theoretical reference for the improvement of CMS-D2 cytoplasm in three-line hybrid cotton breeding.

## Materials and methods

### Plant materials and growth conditions

In our previous study, maintainer line ZB along with its isonuclear alloplasmic near-isogenic restorer line ZBR were utilized to investigate pollen fertility under HT stress (Zhang et al., 2020). In this study, our breeding group hybridized ZBR and ZB by reciprocal crossing method, screened out one set of isonuclear alloplasmic near-isogenic restorer lines SH [S(*Rf_1_rf_1_*) with the *G. harknessii cytoplasm*] and NH [N(*Rf_1_rf_1_*) with the *Gossypium hirsutum cytoplasm*], and at the same time ZB [N(*rf_1_rf_1_*) with the *G. hirsutum* cytoplasm] self-preservation, rename it NB. All seeds of three materials were then planted in the main cotton-producing areas in inland China such as in Anyang (36°10′N, 114°35′E), Henan province in the Yellow River Basin, and Jiujiang (29°71′N, 115°85′E), Jiangxi Province in the Yangtze River Basin during the year 2020. The desired plant materials were sown in the experimental field following natural conditions and standard field management practices. In late July and early August, pollens were collected in Anyang (AP) and Jiujiang (JP), respectively. Pollen from ten plants was collected as one biological replicate, and a total of six biological replicates were collected. All samples harvested for transcriptomics and metabolomics were frozen in liquid nitrogen and immediately stored at −80°C before further analysis. At the same time, the pollen activity was determined by the benzidine methylnaphthol staining method (Li et al., 2018).

### Metabolite extraction and mass spectrometry analysis

To measure the metabolites in desired pollen samples, the samples were thawed on ice before further use. One gram of each mature pollen sample was used to make powder form. Subsequently, under a 1-min vortex, the sample was extracted using 120 microliters of 50% methanol buffer before manual cooling, and then incubated at ambient temperature for 10 min and stored at −20°C. In the next step, the samples were centrifuged at 4,000 *g* for 20 min. The supernatant liquid was finally transmitted to 96-well plates and stored at −80°C for LC-MS analysis. LC-MS data pre-processing was captured using XCMS software. Metabolites eluted from chromatographic columns were detected using a high-resolution tandem mass spectrometer Tripletof5600 Plus (SCIEX, Cheshire, UK). The raw data files were processed with XCMS, CAMERA, and metaX toolbox implemented in R software. The identification of each ion is obtained by the comprehensive information of retention time-m/z. To explain the physical and chemical properties and biological functions of metabolites, public databases, such as HDBM and KEGG, are used for primary and secondary identification and annotation of candidate substances. The significant difference among metabolites was examined with importance in projection (VIP) value combined with FDR (Benjamini–Hochberg) test. The selection criteria were VIP ≥ 1 along with FDR ≤ 0.5 or ≥ 0.5. In addition, the PLS-DA method in metaX was further used to discriminate specific differences between the different pollen samples.

### Ribonucleic acid extraction, illumine sequencing, and transcriptome data analysis

Total ribonucleic acid (RNA) was extracted and purified using TRIzol reagent (Invitrogen, Carlsbad, CA, USA) from specific pollen samples. The RNase-free DNase I (TaKaRa, Kyoto, Japan) was added to remove genomic DNA contamination. The RNA purity of each sample was measured with NanoDrop ND-1000 (NanoDrop, Wilmington, DE, USA). In addition, the RNA integrity was detected with Bioanalyzer 2100 (Agilent, CA, USA) and confirmed by electrophoresis. Then, the final cDNA libraries for each sample were constructed according to the protocol for the mRNA-Seq sample preparation kit (Illumina, San Diego, CA, USA). After sequenced libraries were prepared with standard quality. The average insert size for the final cDNA library was 300 ± 50 bp in our sequencing. The 2 × 150 bp paired-end sequencing (PE150) was performed on an Illumina Novaseq™ 6000 (LC-Bio Technology Co., Ltd., Hangzhou, China) following the vendor’s recommended protocol. Cutadapt software^1^ was used to eliminate low-quality reads from original sequenced data to obtain high-quality reads. HISAT-2.0.4 was used to map reads to the genome of *G. hirsutum*, HAU.^2^ The stringTie-1.34 with default parameters was applied to assemble mapped reads. The final transcripts were then produced with gffcompare-0.9.8. The StringTie along with a ballgown were used to determine the expression levels and to perform expression levels for mRNAs in the FPKM form. The differentially expressed mRNAs were identified between samples with fold change > 2 or fold change < 0.5 and *P*-value < 0.05 by R package DESeq2. GO enrichment and KEGG enrichment analysis were then performed for differentially expressed mRNAs.

### Gene co-expression network construction and identification of the hub genes

The WGCNA was investigated to identify hub genes by following step by step method as previously described (Shahzad et al., 2020). In brief, the expression matrix of each gene generated different functional modules based on their connectivity and expression trends. The genes having the highest intramodular connectivity in each functional module calculated by the WGCNA algorithm were considered hub genes in this study. DEGs in the blue module were selected to construct a co-expression network with Cytoscape v3.7.0, and their interconnected genes had weights ≥ 0.60. Finally, the DEGs co-expression networks were analyzed by molecular complex detection (MCODE), which divided this given network into different clusters based on the topology to discover densely interconnected regions. The R package models were applied to perform all statistical analyses. The least significant difference (LSD) test at *P* < 0.05 were the criteria of significant differences among various samples. The final heatmaps were generated with TBtools software (Version 1.068) (Chen et al., 2020).

## Results

### Abnormal pollen development in restorer with the cytoplasm of *G. harknessii*

**FIGURE 1:**
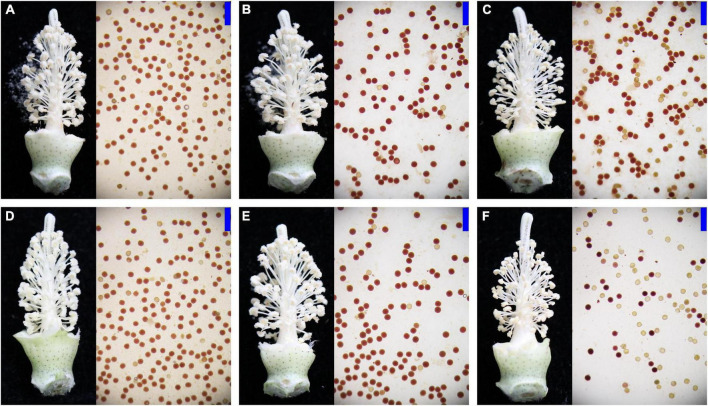
Representative anther phenotypes of SH, NH, and NB in Anyang and Jiujiang. **(A–C)** Pollen from NB, NH, and SH in Anyang. **(D–F)** Pollen from NB, NH, and SH in Jiujiang. The fertile pollen grains were stained dark red and sterile pollen grains were stained bright yellow. Scale bars: 200 μm.

To observe the male infertility of CMS cotton having different cytoplasmic backgrounds, the pollen grains of NB, NH, and SH collected from Anyang and Jiujiang were stained with benzidine methylnaphthol, respectively. Indehiscent anther walls with inactive pollen grains were observed in the SH from Anyang and Jiujiang. Specifically, the proportion of anther indehiscence is higher in Jiujiang, where the temperature is higher. In contrast, the pollen fertility of NH and NB was normal in both environments (Figure 1). The abnormal pollen development in SH compared to NH and NB was most likely produced due to the cytoplasmic effects of *G. harknessii*. These detrimental effects should be investigated to improve hybrid cotton yield under the current scenario of climate change.

### Metabolomic analysis

**FIGURE 2:**
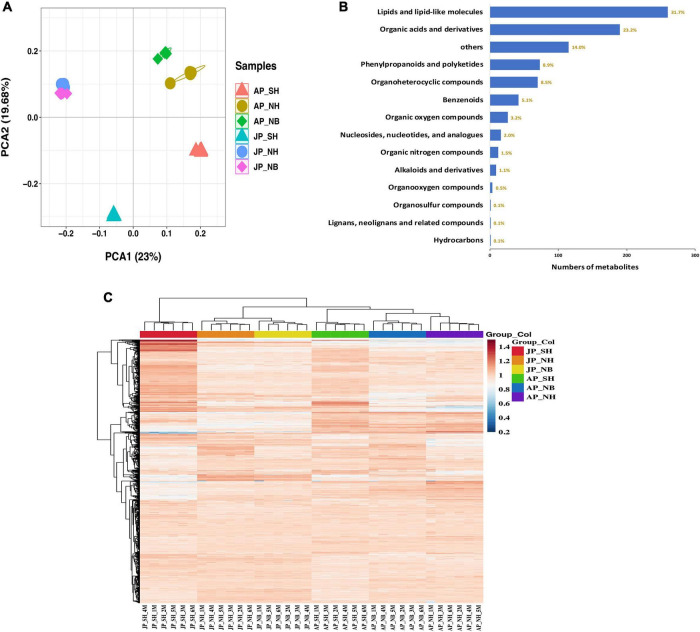
Preliminary analysis of metabolites data. **(A)** Principal component analysis (PCA) score plot metabolite profiles from different groups. **(B)** All metabolite types and quantities. **(C)** The clustering heat maps of all metabolites.

The metabolite profiles of mature pollen fertility from different plant materials were analyzed to identify key metabolites related to pollen fertility. The principal component analysis (PCA) (Figure 2A) based on metabolomics data revealed obvious differences among various sample groups. The PCA1 and PCA2 accounted for 23% and 19.68% of the total variation, respectively. Moreover, a significant differences among different CMS lines in different environments. A total of 820 metabolites were obtained in all samples, of which lipids and lipid-like molecules were the most abundant group, followed by organic acids derivatives, phenylpropanoids, and polyketides (Figure 2B and Supplementary Table 1). All metabolites heatmap showed distinct clusters for each environment and pollen sample (Figure 2C).

**FIGURE 3:**
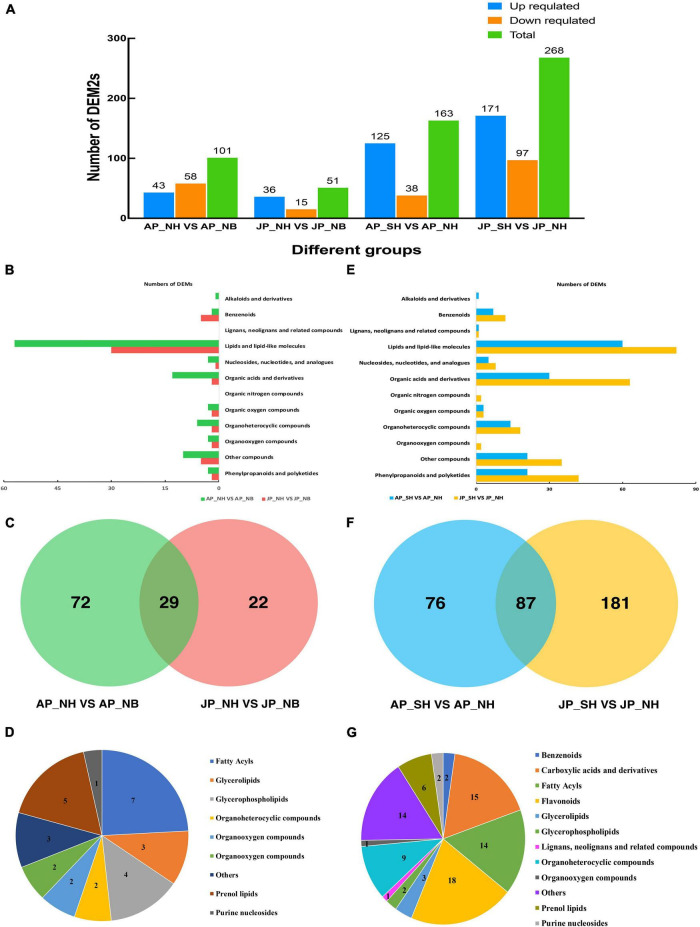
Analysis of differentially expressed metabolites. **(A)** Number of DEMs in different comparison groups. **(B)** The type and quantity of differential metabolites in NH and NB comparison groups under two environments. **(C)** Venn diagram of differential cumulative metabolites in NH and NB comparison group. **(D)** Types and quantities of differential metabolites shared by NH and NB comparison groups. **(E)** The type and quantity of differential metabolites in SH and NH comparison groups under two environments. **(F)** Venn diagram of differential cumulative metabolites in SH and NH comparison group. **(G)** Types and quantities of differential metabolites shared by SH and NH comparison groups.

A total of 101 and 51 differentially expressed metabolites were detected among NH versus NB comparisons in Anyang and Jiujiang, respectively (Figure 3A). The major metabolites were lipids and lipid-like molecules, followed by organic acids and derivatives (Figure 3B). A total of 29 metabolites were common in both Anyang and Jiujiang (Figure 3C). Among them, fatty acyls accounted for the largest proportion of metabolites, followed by prenol lipids, and purine nucleosides (Figure 3D).

In the comparison of SH versus NH, a total of 163 differentially expressed metabolites were detected in Anyang while 268 metabolites showed a significant difference in Jiujiang (Figure 3A). The larger number of differential metabolites in Jiujiang than Anyang suggested dynamic changes in pollen metabolites in Jiujiang. Interestingly, the restorer gene interaction with the sterile cytoplasm of *G. harknessii* caused large differences in lipids and lipid-like molecules, organic acids and derivatives, phenylpropanoids, and polyketides (Figure 3E). However, fewer types of metabolites were identified when the restorer gene is induced alone. The distribution of metabolites in the comparison group of SH versus NH found that a total of 87 metabolites overlapped between Jiujiang and Anyang (Figure 3F). More than half of them belong to flavonoids, carboxylic acids, derivatives, and fatty acyls (Figure 3G). The results suggested that the presence of cytoplasm of *G. harknessii* affects the dynamic balance of lipid metabolism and flavonoid synthesis. This disturbance in metabolic substances mediates pollen development as well as participates in the process of fertility restoration of CMS-D2 cotton.

### Transcriptome analysis

**FIGURE 4:**
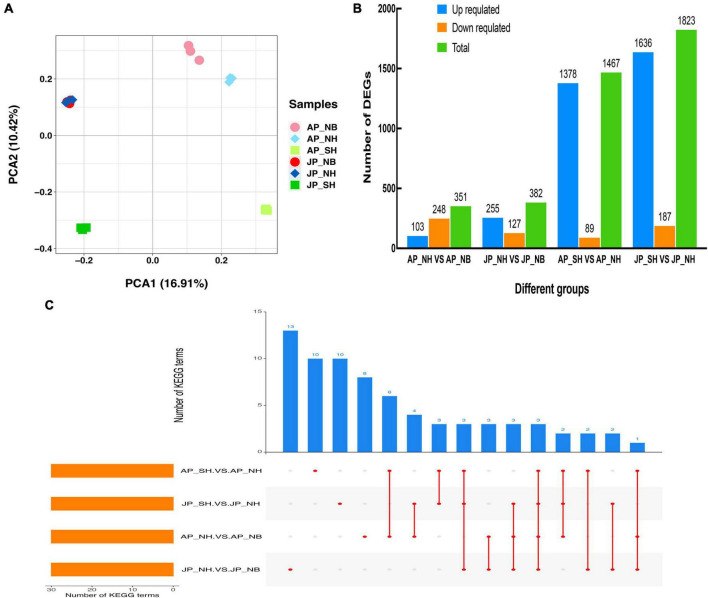
**(A)** Principal component analysis (PCA) score plot genes profiles from different groups. **(B)** Number of differentially expressed genes (DEGs) in different comparison groups. **(C)** The number of KEGG pathways of DEGs in different comparison groups.

In total, eighteen cDNA libraries (six samples × three replications) were constructed for RNA sequencing by utilizing pollen samples named AP_NB, AP_NH, AP_SH, JP_NB, JP_NH, and JP_SH. It yielded approximately 6.3 Gb of sequence data for each library. The brief detail of the RNA-seq data for all 18 libraries is presented in Supplementary Table 2. Each library consisted of more than 33, 431, 830 valid reads. The GC contents ranged from 44.0% to 45.0%, and the scores for each library were greater than 98.17%, indicating the reliability and high quality of the sequencing data obtained in this study. In addition, ensures the accuracy of sequence assembly and coverage of the transcriptome. Principal component analysis (PCA) of the samples revealed replicates of each pollen sample clustered together that indicating the high reliability of our sequencing results (Figure 4A). The PC1 exhibited a 16.91% variance in the data and represented that the difference between SH versus NH was large in Anyang or Jiujiang whereas the difference between NH versus NB was comparatively small. A total of 351 and 382 DEGs were detected in the comparison of SH versus NH in Anyang and Jiujiang, respectively. Moreover, the total number of DEGs in the SH versus NH was significantly higher than that NH versus NB comparison group. Specifically, the upregulated DEGs in both Anyang and Jiujiang were in large numbers in SH versus NH (Figure 4B). Most of the DEGs were stable in the NH versus NB showing significantly upregulated expression in the SH versus NH. The comparative analysis indicates that more dynamic changes were detected in SH versus NH in both environments. KEGG analysis was performed for all four pairwise groups (Figure 4C and Supplementary Table 3). The analysis had shown that cysteine and methionine metabolism, carbon fixation in photosynthetic organisms, and nitrogen metabolism pathways were enriched simultaneously in all comparative groups. Notably, pathway enrichment analysis in AP_SH/AP_NH and JP_SH/JP_NH determined that most genes had shown enrichment in the biosynthesis of unsaturated fatty acids, phenylalanine metabolism, and phagosome. Consistent with the result of the metabolomic analysis, the disruption of the dynamic balance of lipid metabolism may be the main factor leading to the change in pollen fertility.

### Co-expression network construction to identify hub genes

**FIGURE 5:**
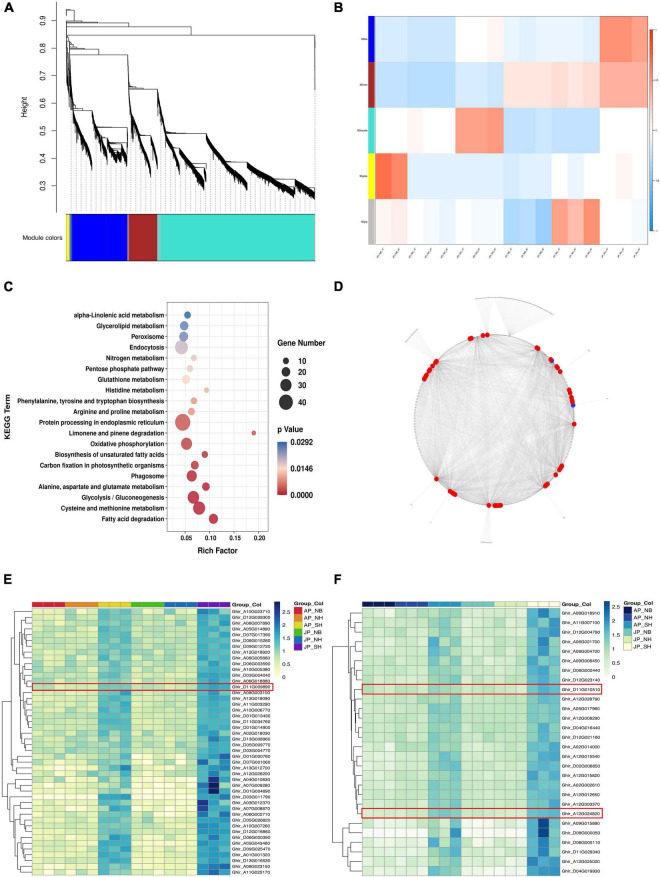
**(A)** Overview of a weighted correlation network analysis based on differentially expressed genes (DEGs). **(B)** The module–sample relationships. **(C)** Pathways of enriched genes in blue module. **(D)** Co-expression network of genes in the blue module. The red node represents the expression pattern of the hub gene in cluster 1, and the blue node represents the expression pattern of the hub gene in cluster 2. **(E,F)** The clustering heat maps of hub genes in cluster 1 **(E)** and cluster 2 **(F)**.

The weighted correlation network analysis of the DEGs was investigated to identify the gene regulatory network of pollen fertility of CMS-D2 cotton (Figure 5A). The expression profiles of DEGs from 17 samples were used to perform co-expression network analysis, with samples AP_NB_3T as outliers. It became clear that each module had higher overlaps so the network was divided into five modules. The expression trend of genes in the blue module tends to be consistent among the three materials under two environmental conditions The expression levels of the blue module genes had a similar trend in NH and NB but were significantly upregulated in SH (Figure 5B). KEGG enrichment analysis for DEGs in the blue module had shown that fatty acid degradation, phenylalanine, tyrosine, tryptophan biosynthesis, and alpha-Linolenic acid metabolism were enriched simultaneously (Figure 5C). In addition, a total of two clusters of the DEG (weight ≥ 0.6) co-expression network were detected by MCODE. The DEGs with the highest intramodular connectivity were considered hub genes from each cluster. It was observed that cluster 1 consisted of 46 hub genes whereas cluster 2 had 28 hub genes (Figure 5D). To investigate how hub genes responded to the cytoplasm of *G. harknessii*, we examined the expression profiles of hub genes, all of which were consistently expressed in comparison groups of SH versus NH in different environments (Figures 5E,F). Further analysis of these genes, we identified three transcription factors, including *WRKY22* (*Ghir_D11G009890*), *WRKY53* (*Ghir_A12G024820*), and *ARF18* (*Ghir_D11G010510*). These transcription factors were significantly upregulated in SH in Anyang and Jiujiang, suggesting that these centers may play a key role in pollen development.

### Lipid metabolism influence pollen fertility of different cytoplasmic lines

**FIGURE 6:**
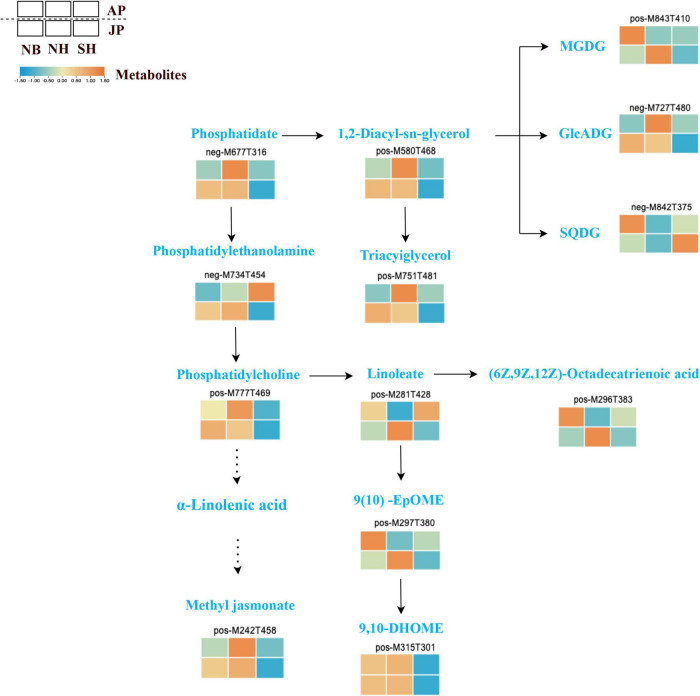
Different expressions of structural metabolites in lipid metabolism. Red and blue blocks represent upregulation and downregulation of expression, respectively.

Here, differential genes and metabolites related to lipid metabolism were identified in NB, NH, and SH under both environmental conditions. Further in-depth analysis of the biological pathways of lipid biosynthesis was examined by integration of metabolomics and transcriptomics data. A dynamic difference in this pathway, the abundance of lipid compounds are shown in Figure 6. Lipid metabolism mediates sexual reproduction in plants and the destruction in lipid composition during pollen development often leads to pollen infertility and even male sterility. In this study, we found that some metabolites in the glycerolipid metabolism pathway had shown significant downregulation in SH. For example, MGDG (pos-M843T410) and GICADG (neg-M727T480), as well as prerequisites for glycerol synthesis, phospholipids (neg-M677T316) and 1,2-diacyl-sn-glycerol (POS-M580T468), which are prerequisites for the synthesis of glycerols, are also showed significant downregulation. Moreover, some unsaturated fatty acids showed significant downregulation in SH, such as 9(10)-EpOME (pos-M297T380), 9-10-DHOME (pos-M315T301), and (62,9Z,12Z)-Octadecatrienoic acid (pos-M296T383). In the jasmonic acid synthesis pathway, most of the unsaturated fatty acids were downregulated in SH in both environments. In addition, phosphatidylcholine (pos-M777T469) was involved in the formation of α-linolenic acid, and methyl jasmonate had shown downregulation in SH. The significant downregulation of metabolites in SH most probably breaks the balance of lipid metabolism, which may be the main factor leading to the decline of pollen fertility in SH. These results suggest that metabolites tightly linked with lipid and jasmonate play a critical role in pollen fertility of CMS-D2 cotton.

### Flavonoid biosynthesis influences pollen fertility of different cytoplasmic lines

**FIGURE 7:**
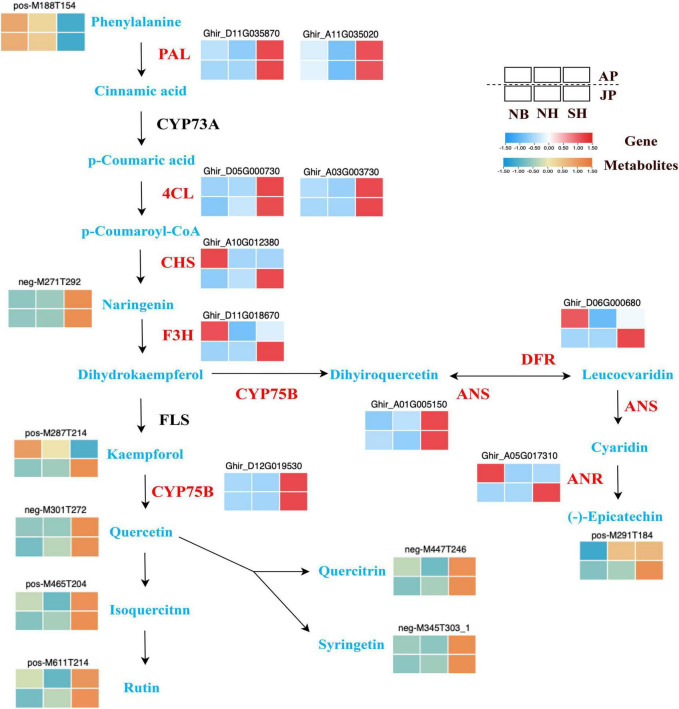
Different expressions of structural genes (red) and metabolites in flavonoid biosynthesis pathway. Red and blue blocks represent upregulation and downregulation of expression, respectively. Abbreviations: PAL, phenylalanine ammonia lyase; 4CL, 4-eoumarate coenzyme A ligases; CHS, chalcone synthase; F3H, flavonoid 3-hydroxylase; CYP75B, cytochromes P450; ANS, anthocyanidin synthase; DFR, dihydroflavonol 4-reductase; ANR, anthocyanidin reductase.

Our analysis through the integration of metabolomics with transcriptomic analyses further identified dynamic changes in flavonoid biosynthesis pathways among different materials and environments. The regulatory correlation among DEGs and the abundance of key flavonoid compounds are presented in Figure 7 for NB, NH, and SH. At the initial step of the phenylpropanoid pathway, phenylalanine ammonia-lyase (*PAL*) facilitates the biosynthesis of cinnamic acid. Then, 4-Eoumarate coenzyme A ligases (*4CL*) contribute to the generation of coenzyme A (CoA) esters during the biosynthesis of hydroxycinnamic acid. In this study, several *PAL* and *4CL* encoding genes had shown higher levels of expression in the SH pollen samples under both environmental conditions. The chalcone isomerase (CHI) realted genes convert chalcones into naringenins or flavanones. While flavanone 3-hydroxylase (F3H) annotated genes influenced the hydroxylation of flavanones to generate dihydrokaempferol. The genes associated with CHS and FEH along with flavonol synthase (FLS) are important structural genes of the flavonoid biosynthesis pathway. These genes transferred p-coumaroyl CoA into kaempferol. Interestingly, the cytochromes P450 (CYP75B) that catalyzes the production of flavanonols from kaempferol is upregulated in both environments. In addition, a higher accumulation of flavonols (quercetin, quercitrin, syringetin) was determined in this study. This may be caused by the upregulation of the CYP75B genes. Moreover, genes associated with mRNAs encoding flavonoid 3’-monooxygenase (*CYP75B*) and anthocyanidin synthase (*ANS*) were all upregulated in SH than in NH under two environmental conditions. It may be the main reason for the excessive accumulation of (-)-Epicatechin. In brief, DEGs and metabolites closely correlated with flavonoid synthesis possibly responsible for the observed pollen differences between SH and NH. Therefore, an in-depth analysis of genes related to flavonoid synthesis will be essential to understanding the potential molecular mechanism of pollen sterility and fertility in CMS-D2 cotton.

## Discussion

Cytoplasmic male sterility is critical to exploit heterosis in three-line hybrid cotton breeding programs. The cytoplasm causes substantial genetic effects on hybrid progeny and is thus essential for yield improvement enhancement in cotton breeding. Despite its economic value, CMS-D2 has negative effects on yield-related traits in cotton (Meyer, 1973; Weaver, 1986; Zhang et al., 2019a). In addition, more and more studies have shown that high temperature often leads to decreased pollen fertility, anther non-dehiscence, and so on, pollen fertility of CMS-D2 F_1_ hybrids is more prone to temperature stress than normal upland cotton F_1_ hybrids. High-temperature stress at the flowering stage decreased the pollen fertility of CMS-D2, and significantly reduced the number of bolls per plant, especially in the Jiujiang cotton belt of the Yangtze River cotton valley (Zuo et al., 2022). Therefore, the decline in pollen fertility is most probably the direct reason for the decline in cotton yield and quality. This study through the integration of metabolomics and transcriptome compared the differences in pollen fertility of different cytoplasmic backgrounds CMS lines in the two environments. In the presence of restorer genes, the number of differential metabolites and genes identified in CMS-D2 cytoplasm was significantly larger than in normal upland cotton cytoplasm. Moreover, with the higher temperature in the Yangtze River basin, the number of differential metabolites and negative effects in Jiujiang is more obvious, which is consistent with previous reports (Ni et al., 2009). Based on the combined analysis of metabonomics and transcriptome, our study found that the pathways of lipid metabolism and flavonoid synthesis in sterile cytoplasm were different from those in fertile cytoplasm. Therefore, further discussed their key role in pollen fertility of CMS-D2 cotton.

Lipids are an important integral part of the plant, contribute to different metabolic activities, and are crucial for reproductive development in plants. The interruption of lipid metabolism balance often leads to the decline of pollen fertility as well as disorder leads to anther dehiscence, pollen immaturity (Ishiguro et al., 2001; Xiao et al., 2014), and pollen hydration (Fiebig et al., 2000; Haslam et al., 2015; Zhan et al., 2018). In this study, many lipids-related metabolites were identified to be significantly downregulated in both environments. In particular, most of the downregulated substances, especially unsaturated fatty acids, including α-linolenic acid and linoleic acid synthesis precursor phosphatidylcholine, decreased significantly in SH with sterile cytoplasm compared with NH. The decrease of these unsaturated fatty acids only existed in the restorer line with sterile cytoplasm, but there were no significant changes between NH and NB with the normal upland cotton cytoplasm (Figure 6). This finding suggests that the sterile cytoplasm may affect pollen fertility by disrupting lipid metabolism as consistent with the earlier research studies (Fu et al., 2015; Khan et al., 2020). Jasmonic acid (JA) is widely distributed in plants and is derived from α-linolenic acid (He et al., 2021). JA along with its derivatives play important role in the regulation of many physiological aspects of plant growth and development (Ruan et al., 2019). Furthermore, it influenced anther dehiscence, filament elongation, and pollen viability (Scott et al., 2004). Interestingly, this study determined a significant decline in methyl jasmonate in both environments for SH. This reduction in the biosynthesis of jasmonate most likely causes a decline in pollen viability. Thus, we deduced that JA synthesis and signal transduction pathways could play pivotal roles in pollen development. Additionally, co-expression network analysis revealed 74 hub genes, including two *WRKY* transcription factors *WRKY22*, *WRKY53*, and one auxin response factor *ARF18*, which had a tight relationship with pollen fertility (Figures 5E,F). Earlier studies stated that *WRKY22* most likely contributes to anther/pollen development by inhibiting the expression of the JA signaling pathway, and overexpression of *GhWRKY22* in Arabidopsis can reduce pollen viability and germination rate (Wang et al., 2019). *WRKY22* and *WRKY53* are also involved in biological and abiotic stresses. *WRKY22 i*s a suppressor of JA signaling in response to aphid induction (Kloth et al., 2016, 22), and *WRKY53* plays a role in plant defense through negative regulation of the *Arabidopsis* jasmonate biosynthesis pathway (Hu et al., 2015, 53; Jiao et al., 2022, 53). In this study, *WRKY22* and *WRKY53* were significantly upregulated in SH compared with NH in both environments. However, the NH and NB comparison groups had shown no significant difference in the two environments (Figures 5E,F). These results revealed that upregulated expression of *WRKY22* and *WRKY53* inhibited the synthesis of jasmonate, consistent with the downregulation of methyl jasmonate in our metabolomic data. This may be the main reason for the decline in pollen fertility of SH with the sterile cytoplasm. JA feeding studies and JA measurements indicate that the *ARF* transcription factor regulated anther dehiscence by regulating JA synthesis or signal transduction (Wang et al., 2013). Another research has reported that *ARF6* and *ARF8* mutants in *Arabidopsis* showed delayed stamen development and decreased fecundity (Nagpal et al., 2005). Considering *ARF18* here was upregulated in SH compared with NH in both environments (Figure 5F), therefore we speculate that it may be a negative regulator of pollen fertility by activating the auxin signaling pathway (Ding et al., 2017).

Flavonoids are plant secondary metabolites that include monomeric flavonols, flavanones, flavones, and flavonols. These are the functional parts of many biological processes in plants. Previous studies have confirmed the importance of flavonoids for plant fertility and sexual reproduction, especially in pollen development, pollen germination, or pollen tube growth in rice (Wang et al., 2020), maize (Mo et al., 1992), cotton (Kong et al., 2020), petunia (Taylor and Jorgensen, 1992), and tomato (Paupière et al., 2017). In addition, flavonoids are one of the powerful secondary metabolites that defend plants against ROS stress production during stresses (Rice-Evans et al., 1996). In plants, flavonoids can be oxidized by POD and contribute to scavenging H_2_O_2_ in the phenolic/AsA/POD system (Sharma et al., 2012). Exogenous application of flavonol can accelerate their fertility restoration function in plants. Specifically, flavonol significantly reduces the abundance of ROS in pollen and reduces the negative effects on pollen viability and germination during heat stress (Muhlemann et al., 2018). According to many previous research reports, abnormal ROS status is closely linked with pollen abortion in various CMS plants, excessive accumulation of ROS in cytoplasmic male sterile lines, and the expression of flavonoids in male sterile lines is significantly downregulated compared with maintainer lines, which cannot effectively remove too much ROS and lead to programmed cell death, resulting in pollen fertility (Deng et al., 2012; Ding et al., 2019; Zhang et al., 2019b). Recent research on CMS-D2 demonstrated that the cotton mitochondrial chimeric gene *orf610a* disturbs the dynamic balance between ATP synthesis and ROS outbreak (Zhang et al., 2022). In this study, a large number of genes and metabolites annotated with flavonoid biosynthesis were upregulated in the sterile cytoplasm. The phenylalanine ammonialyase (PAL), cinnamic acid hydroxylase (C4H), and coumarin CoA ligase (4CL) catalyzed the phenylalanine and in this way initial product p-Coumaroyl-CoA of flavonoid synthesis. The PAL and 4CL were significantly upregulated in SH pollen in both environments. Besides this, naringenin is the common precursor of many intermediate metabolites as well as it drives the structural similarity among flavonoids. In addition, under the influence of flavonol synthase (FLS), dihydroflavonol produced from naringenin later synthesizes various flavonols such as kaempferol, quercetin, and quercitrin. All these flavonols were significantly enriched in the sterile cytoplasm. Concisely, the flavonoid metabolites encoding genes along with restoring gene *Rf*_1_ influenced pollen development in the sterile cytoplasm and involves in the process of fertility restoration. These genes most probably facilitate the clearance efficiency of ROS through the flavonoid synthesis pathway thus achieving fertility restoration. However, compared with the fertile cytoplasm, the fertility restoration is still incomplete, and pollen fertility may affect by the sterile cytoplasm in other ways. Pollen fertility restoration is a complex regulatory process in CMS cotton. This specific mechanism needs to be further studied.

**FIGURE 8:**
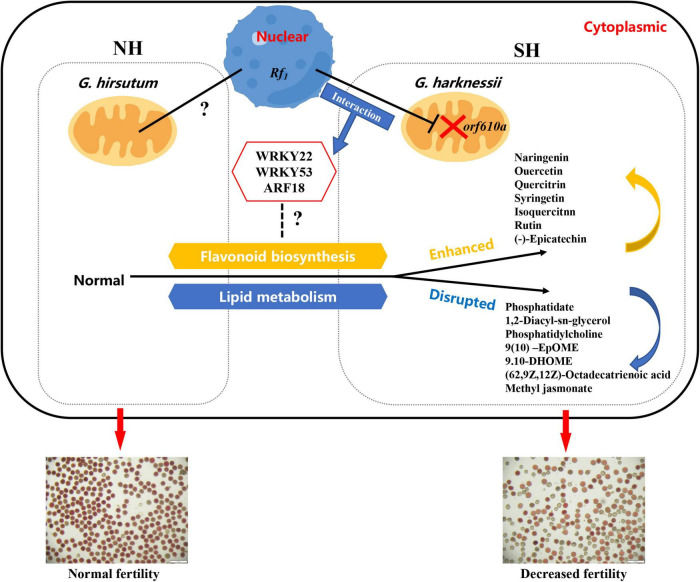
A proposed model showing the main factors affecting pollen fertility of restorer lines with different cytoplasms in the presence of restorer gene *Rf*_1_.

Taken together, here we propose a potential regulatory model of how the sterile cytoplasm of CMS-D2 affects the pollen fertility in cotton (Figure 8). For the upland cotton cytoplasmic restorer line NH, lipid metabolism balance and flavonoid synthesis homeostasis synergistically ensure normal pollen development. In comparison, in the presence of CMS-D2 sterile cytoplasm, the cytoplasmic-nucleus interaction effects may enhance flavonoid synthesis involved in the fertility restoration process and induce the upregulated expression of key transcription factors such as *WRKY22*, *WRKY53*, and *ARF18*, which disrupts the balance of downstream lipid metabolism and ultimately lead to the decline of pollen fertility in restorer SH, but the specific regulatory mechanism is still unclear.

## Conclusion

In conclusion, this study provides a comprehensive analysis of the molecular mechanism of the cytoplasmic effects of CMS-D2 on pollen fertility. Based on WGCNA, some key hub genes, such as *WRKY22*, *WRKY53*, and *ARF18* have been identified, which may be involved in the regulation of pollen fertility. These hub genes influence the synthesis of unsaturated fatty acids. The negative effect of CMS-D2 cytoplasm on pollen is mainly due to breaking the lipid metabolism, especially the metabolic balance of unsaturated fatty acids, finally resulting in the decline of pollen fertility. This situation is more obvious under HT stress. In the presence of restoring gene, the process of fertility restoration will induce a large amount of flavonoid synthesis, which promotes the restoration of pollen fertility. These findings can help elucidate the molecular mechanism and regulatory network of the effect of male-sterile cytoplasm on pollen fertility and provide a biological basis for the improvement of CMS-D2 cytoplasm.

## Data availability statement

The clean data presented in this study have been deposited in the National Center for Biotechnology Information (NCBI) under Sequence Read Archive (SRA) accession number: PRJNA875235, available at https://www.ncbi.nlm.nih.gov/bioproject/PRJNA875235.

## Ethics statement

All the cotton lines used and analyzed were public and available for non-commercial purposes. This article did not contain any studies with human participants or animals performed by any of the authors.

## Author contributions

CX and JW designed the experiments. LG, TQ, HT, XQ, RZ, and HW performed the field management. XZ, MZ, YL, JF, and ZZ performed data analysis and phenotypic identification. XZ, MZ, and KS contributed to the preparation of the manuscript. All authors read and approved the final manuscript.

## Footnotes

1 https://cutadapt.readthedocs.io/en/stable/, version: cutadapt-1.9.

2
https://cottonfgd.net/about/download.html

## Supplementary material

Supplementary Table 1 The information on metabolomes.

Supplementary Table 2 Summary statistics of sequence data.

Supplementary Table 3 KEGG pathway analysis of differentially expressed genes (DEGs).
